# Transferrin Enhances Antifungal Therapy and Improves Survival in Experimental Mucormycosis

**DOI:** 10.3390/jof12090682

**Published:** 2026-09-11

**Authors:** Yiyou Gu, Teclegiorgis Gebremariam, Belal A. Ibrahim, Keenan Harb, Andrew Chan, Ashraf S. Ibrahim

**Affiliations:** 1 Division of Infectious Diseases, The Lundquist Institute for Biomedical Innovation, Harbor-University of California at Los Angeles (UCLA) Medical Center, Torrance, CA 90502, USA; yiyou.gu@lundquist.org (Y.G.);; 2 Vitalex Biosciences LLC, Trabuco Canyon, CA 92678, USA; 3 Department of Medicine, David Geffen School of Medicine at UCLA, Los Angeles, CA 90095, USA

**Keywords:** mucormycosis, transferrin, antifungal

## Abstract

Mucormycosis is a highly lethal invasive fungal infection caused by fungi of the order Mucorales, with mortality rates exceeding 50% despite current antifungal therapies with polyenes or azoles. Because iron acquisition is essential for Mucorales growth and virulence, we investigated whether transferrin, the primary physiological iron-sequestering protein in plasma, could serve as a host-directed therapeutic strategy against mucormycosis. Transferrin inhibited the growth of clinically relevant Mucorales species, including *Rhizopus delemar* (*R. delemar*), *Rhizomucor*, and *Lichtheimia corymbifera* (*L. corymbifera*) in a concentration-dependent manner. Checkerboard assays demonstrated synergistic interactions between transferrin and liposomal amphotericin B (LAMB), with fractional inhibitory concentration index values below 0.5 for all tested species. In endothelial cell infection model, transferrin significantly reduced expression of the fungal invasion ligand spore coat protein homolog 3 (CotH3) and the host receptor glucose-regulated protein 78 (GRP78), key mediators of angioinvasion during mucormycosis. In a neutropenic murine model of pulmonary mucormycosis, transferrin enhanced the therapeutic efficacy of both LAMB and isavuconazole (ISAV), resulting in improved survival compared with antifungal monotherapy. Collectively, these findings provide preclinical evidence that transferrin modulates pathogenic determinants associated with mucormycosis and enhances the efficacy of current antifungal therapies, supporting further investigation of transferrin as a potential host-directed adjunctive treatment.

## 1. Introduction

Mucormycosis is one of the most lethal invasive fungal infections, with reported mortality rates ranging from 50% to nearly 100% depending on the site of infection, extent of disease, and underlying host factors [1,2,3,4,5]. The disease is caused by fungi belonging to the order Mucorales, with the most common genera contributing to the infection including *Rhizopus delemar* (*R. delemar*), *Mucor circinelloides* (*M. circinelloides*), *Lichtheimia corymbifera* (*L. corymbifera*), and *Rhizomucor* [6]. Patients with hematologic malignancies, prolonged neutropenia, hematopoietic stem cell transplantation, solid organ transplantation, uncontrolled diabetes mellitus, and other forms of immunosuppression are at particularly high risk for infection [7,8]. Recent expert consensus has also emphasized the importance of identifying high-risk populations and implementing early diagnostic and preventive strategies for mucormycosis, particularly following respiratory viral infections [9]. Despite advances in antifungal therapy and supportive care, clinical outcomes remain poor, highlighting the need for novel therapeutic strategies [10,11,12].

A defining feature of mucormycosis is its marked propensity for angioinvasion [13]. Following germination, fungal hyphae invade endothelial cells and blood vessels, resulting in thrombosis, tissue infarction, hemorrhage, and extensive tissue necrosis [10,14]. Vascular invasion also impairs delivery of immune cells and antifungal agents to infected tissues, further contributing to treatment failure and disease progression [10,15,16]. Molecular studies have identified the interaction between the fungal ligand spore coat protein homolog 3 (CotH3) and the host endothelial receptor glucose-regulated protein 78 (GRP78) as a critical mechanism mediating endothelial cell invasion and vascular injury during mucormycosis [17,18].

Iron acquisition represents another central virulence determinant of Mucorales [15]. The availability of free iron strongly influences fungal growth, germination, and pathogenicity, and conditions associated with increased iron availability, including diabetic ketoacidosis, are well-established risk factors for mucormycosis [7]. Consequently, host mechanisms that restrict iron availability play an important role in innate defense against Mucorales infection.

Transferrin is the major physiological iron-binding protein in plasma and serves as a key component of nutritional immunity by limiting extracellular iron availability [19]. Previous studies have demonstrated that transferrin-mediated iron sequestration can inhibit the growth of bacterial and fungal pathogens [19]. In addition to its role in iron restriction, emerging evidence suggests that modulation of iron availability may influence fungal virulence pathways that contribute to tissue invasion and disease progression [20].

Current treatment of mucormycosis relies primarily on antifungal therapy with liposomal amphotericin B (LAMB) or isavuconazole (ISAV) [21]. Despite the availability of these agents, mortality remains unacceptably high, highlighting the need for adjunctive therapeutic strategies that improve treatment efficacy. Because transferrin restricts iron availability and may influence fungal virulence, we hypothesized that transferrin could augment antifungal activity and improve therapeutic outcomes during mucormycosis.

In the present study, we evaluated the antifungal activity of transferrin against clinically relevant Mucorales fungi and examined its interactions with clinically used antifungal agents. We demonstrate that transferrin inhibits fungal growth, synergizes with LAMB in vitro, suppresses expression of CotH3 and GRP78, and significantly enhances survival when combined with antifungal therapy in a neutropenic murine model of pulmonary mucormycosis. Importantly, transferrin improved therapeutic efficacy when combined with both LAMB and ISAV, supporting further preclinical investigation of transferrin as a potential adjunctive therapy for mucormycosis.

## 2. Materials and Methods

### 2.1. Fungal Strains and Growth Conditions

*R. delemar* 99-880 and *Rhizomucor* were obtained from the Fungus Testing Laboratory at the University of Texas Health Science Center at San Antonio. *L. corymbifera* strain was recovered from a patient enrolled in the DEFEAT Mucor clinical trial [22]. Fungal isolates were maintained on potato dextrose agar (PDA, Becton Dickinson, Franklin Lakes, NJ, USA, Cat: DF0013-07-8) plates at 37 °C. Fresh spores were harvested by washing cultures with sterile phosphate-buffered saline (PBS, Fisher Scientific, Waltham, MA, USA, Cat: MT21040CV) containing 0.01% Tween-20 (Fisher Scientific, Waltham, MA, USA, Cat: BP337-100) and filtered through sterile cell strainers (Fisher Scientific, Waltham, MA, USA, Cat: 22-363-547) to remove hyphal fragments. Spore concentrations were determined using a hemocytometer (Fisher Scientific, Waltham, MA, USA, Cat: 02-671-51B) and adjusted to the desired inoculum for each experiment.

### 2.2. Growth Inhibition Assays

To evaluate the antifungal activity of transferrin, fungal spores were incubated in RPMI 1640 medium (Fisher Scientific, Waltham, MA, USA, Cat: A1049101), supplemented with increasing concentrations of human transferrin (up to 50 μg/mL, Sigma-Aldrich, Chicago, IL, USA, Cat: T1147-500MG) in 96-well microplates (Fisher Scientific, Waltham, MA, USA, Cat: 07-200-103). Following overnight incubation at 37 °C, fungal growth was quantified by measuring optical density at 450 nm (OD450) using a microplate reader (BioTek Synergy2, BioTek Instruments, Inc., Winooski, VT, USA). Growth was normalized to untreated controls, and results were expressed as percent inhibition relative to control cultures. The transferrin concentrations were selected to characterize concentration-dependent antifungal activity under controlled in vitro conditions and were substantially below normal circulating transferrin concentrations in humans. The growth inhibition assay was performed in two independent experiments, with one measurement per condition in each experiment. The mean OD450 value from the two independent experiments is presented for each transferrin concentration and fungal isolate.

### 2.3. Checkerboard Synergy Assays

The interaction between transferrin and LAMB (Astellas Pharma US, Inc., Northbrook, IL, USA, NDC: 0469-3051-30) was evaluated using a standard checkerboard microdilution assay. Serial two-fold dilutions of transferrin and LAMB were prepared in 96-well microplates containing fungal spores in RPMI 1640 medium (Fisher Scientific, Waltham, MA, USA, Cat: A1049101). Plates were incubated at 37 °C for 24 h, and fungal growth was assessed spectrophotometrically. A separate checkerboard assay was performed for each fungal isolate, with each isolate evaluated in a single experiment and one measurement obtained for each concentration combination. The checkerboard concentration ranges were selected to evaluate the interaction between transferrin and subinhibitory concentrations of LAMB and were not intended to independently establish the complete single-agent minimum inhibitory concentration (MIC) range. Single-agent MICs of transferrin and LAMB were determined separately for each fungal isolate and are presented in Supplementary Figure S1.

The interaction between transferrin and LAMB was evaluated using the fractional inhibitory concentration index (FICI), calculated as follows:

FICI = (MIC of transferrin in combination/MIC of transferrin alone) + (MIC of LAMB in combination/MIC of LAMB alone).

Because the single-agent MIC of transferrin exceeded the highest concentration tested (>50 μg/mL), the highest tested concentration was used to calculate an upper-bound FICI, which is reported using the ‘<’ symbol.

The checkerboard FICI approach was selected because it is a commonly used quantitative method for evaluating in vitro antifungal drug interactions and allows combination effects to be classified using established interpretive criteria. Synergy was defined as FICI < 0.5, indifference as FICI ≥ 0.5 to ≤4.0, and antagonism as FICI > 4.0 [23].

The checkerboard results and corresponding FIC/FICI values are summarized in Supplementary Table S1.

### 2.4. Endothelial Cell Infection and Gene Expression Analysis

Human umbilical vein endothelial cells (HUVECs) were cultured in endothelial growth medium under standard conditions [24]. Confluent monolayers were infected with or without *R. delemar* in the presence or absence of human transferrin (10 μg/mL). After 4 h of incubation, total RNA was isolated separately from fungal and host cells using the protocol previously described by our group [25].

cDNA was synthesized using a reverse transcription kit (Fisher Scientific, Waltham, MA, USA, Cat: 4368814), and quantitative real-time PCR (qPCR) was performed to determine expression levels of fungal *cotH3* and host *grp78*. Gene expression was normalized to appropriate housekeeping genes and analyzed using the comparative Ct method. The qPCR analysis was performed in four independent experiments, with each sample analyzed in technical duplicate.

### 2.5. Murine Model of Pulmonary Mucormycosis

All animal procedures were approved by the Institutional Animal Care and Use Committee and performed in accordance with institutional guidelines. Male Institute of Cancer Research (ICR) mice (20 to 23 g) obtained from Envigo (Indianapolis, IN, USA) were used in a neutropenic mouse model of invasive pulmonary mucormycosis [26]. Neutropenia was induced using cyclophosphamide (200 mg/kg given intraperitoneally [i.p.], Sandoz Inc., Princeton, NJ, USA, NDC: 0781-3233-94) and cortisone acetate (500 mg/kg, given subcutaneously [s.c.], Sigma-Aldrich, Chicago, IL, USA, Cat: C3130-5G) on days −2 and +3, relative to infection. Mice were infected intratracheally with 2.5 × 10^5^ *R. delemar* spores suspended in 25 µL PBS. Treatment was initiated 2 h after infection. Mice received vehicle control (5% Dextrose), transferrin (90 mg/kg), LAMB (5 mg/kg), ISAV (78 mg/kg, Astellas Pharma US, Inc., Northbrook, IL, USA, NDC: 0469-0520-02), or the corresponding combination therapies. Transferrin was administered intravenously at 90 mg/kg based on a previously published dose–response study in which 90 and 270 mg/kg produced similar survival benefits in infected mice, and 90 mg/kg was subsequently selected for efficacy studies [27]. Antifungal treatment consisted of LAMB (5 mg/kg, intravenous [IV], once daily) administered from Day 0 through Day 3 after infection, or ISAV (78 mg/kg, oral, twice daily) administered from Day 0 through Day 6 after infection. The ISAV dose represents the administered prodrug (isavuconazonium sulfate), rather than the equivalent dose of active isavuconazole. The LAMB and ISAV combination studies were conducted from two separate experiments. In the LAMB study, infected mice were assigned to placebo, transferrin, LAMB, or transferrin plus LAMB groups (*n* = 12 per group), with an additional uninfected control group (*n* = 10). In the ISAV study, infected mice were assigned to placebo (*n* = 17), transferrin (*n* = 16), ISAV (*n* = 16), or transferrin plus ISAV (*n* = 16), with an additional uninfected control group (*n* = 10). Animals were monitored daily for 21 days following infection. Survival was recorded and analyzed using Kaplan–Meier survival curves. Mice meeting predefined humane endpoints were euthanized and recorded as deaths for survival analysis.

### 2.6. Statistical Analysis

Data are presented as mean ± standard deviation (SD) unless otherwise indicated. Comparisons between experimental groups were performed using one-way analysis of variance (ANOVA) with appropriate post hoc testing or Student’s *t*-test where applicable. Survival curves were analyzed using the log-rank (Mantel–Cox) test. Hazard ratios and 95% confidence intervals were calculated using the log-rank method. Statistical analyses were performed using GraphPad Prism software (Version 11.0.2). A *p* value < 0.05 was considered statistically significant.

## 3. Results

### 3.1. Transferrin Inhibits the Growth of Clinically Relevant Mucorales Fungi

To determine whether transferrin possesses direct antifungal activity against Mucorales, we evaluated its effect on the growth of multiple clinically relevant species, including *R. delemar*, *Rhizomucor*, and *L. corymbifera*. Fungal spores were incubated with increasing concentrations of human transferrin, and growth was quantified by measuring optical density after overnight incubation.

**Figure 1:**
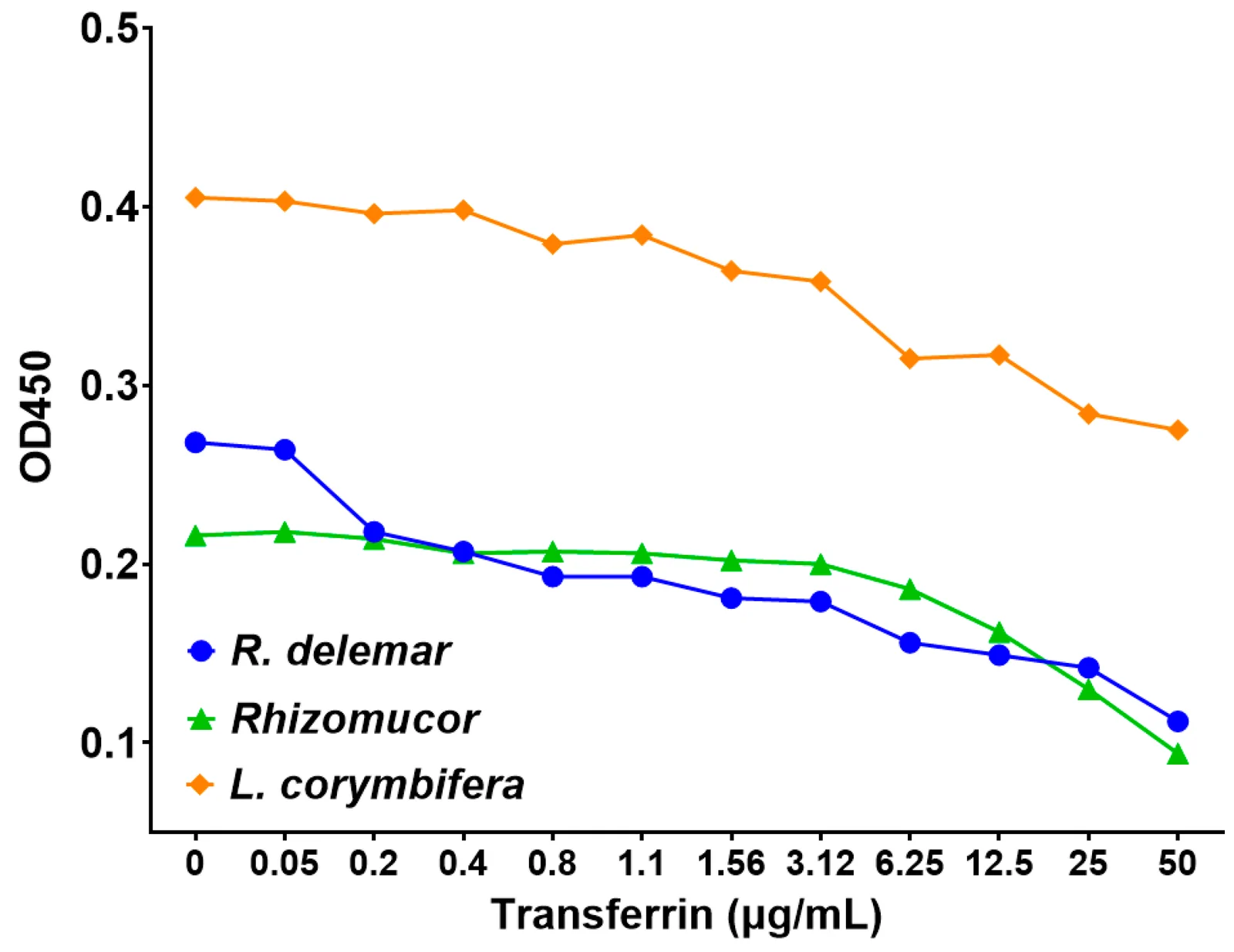
Human transferrin inhibits the growth of clinically relevant Mucorales fungi in a concentration-dependent manner. *R. delemar*, *Rhizomucor*, and *L. corymbifera* were incubated with serial dilutions of human transferrin. Fungal growth was quantified by measuring optical density at 450 nm (OD_450_) following overnight incubation. Data represent the mean OD450 values from two independent experiments, with one measurement per condition in each experiment.

Transferrin inhibited fungal growth in a concentration-dependent manner across all tested species (Figure 1). Although the magnitude of inhibition varied among isolates, all fungi had reduced growth in the presence of transferrin compared with untreated controls. These findings indicate that transferrin exerts antifungal activity against diverse Mucorales fungi and support the concept that iron sequestration may impair fungal proliferation.

### 3.2. Transferrin Synergizes with LAMB Against Mucorales

**Figure 2:**
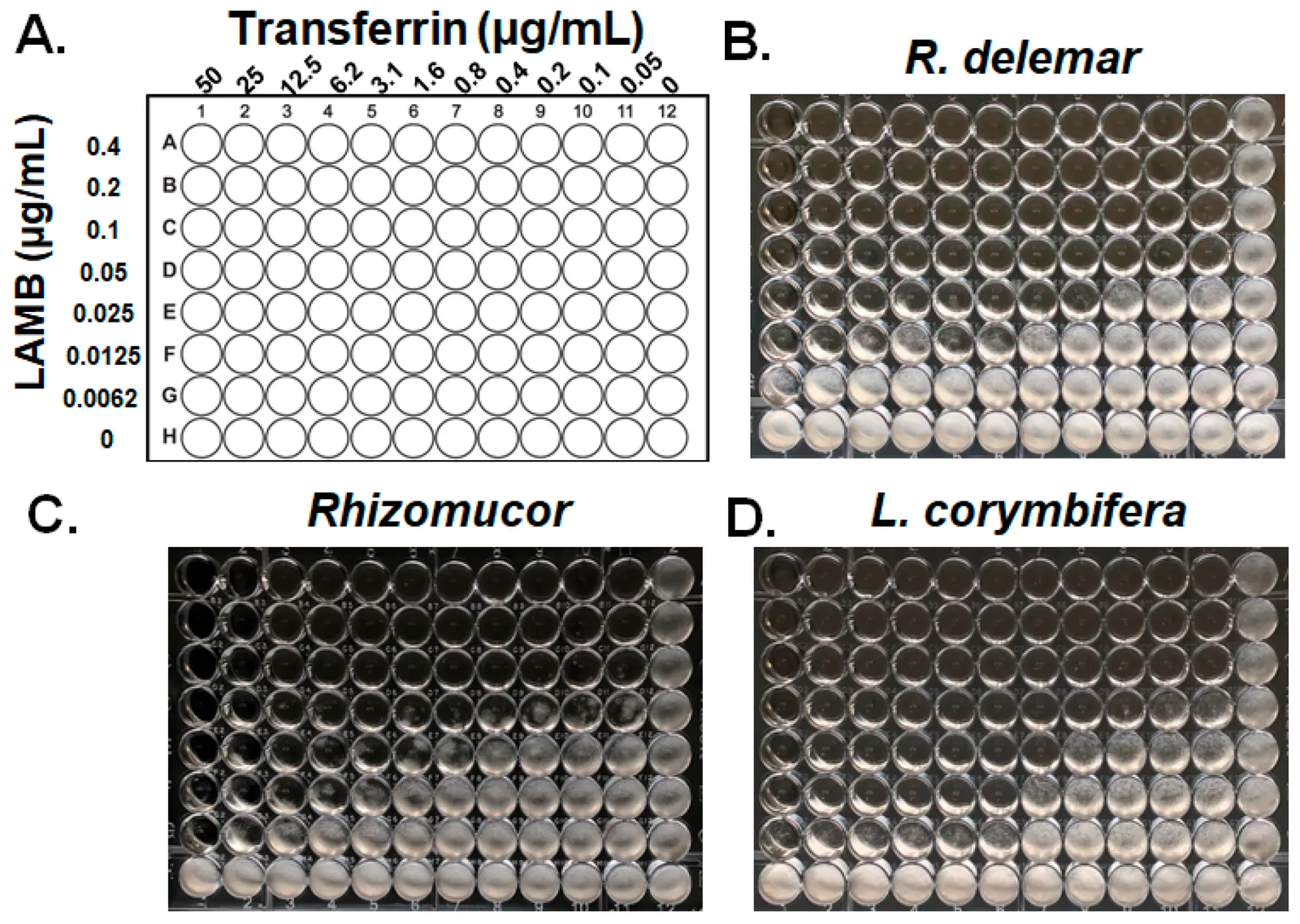
Transferrin exhibits synergistic antifungal activity with LAMB against Mucorales in vitro. (**A**) Experimental design of the fractional inhibitory concentration (FIC) checkerboard assay. Numbers 1–12 indicate decreasing concentrations of transferrin across the columns, whereas letters A–H indicate decreasing concentrations of LAMB across the rows. Representative images of fungal growth in the checkerboard assays are shown for *R. delemar*, *Rhizomucor*, and *L. corymbifera* in (**B**–**D**), respectively. A separate checkerboard assay was performed once for each fungal isolate, with one measurement for each concentration combination.

Because iron availability also regulates virulence-associated pathways in Mucorales, we next investigated whether transferrin influences antifungal activity when used in combination with clinically used antifungal agents. We performed checkerboard fractional inhibitory concentration (FIC) assays using transferrin and LAMB against *R. delemar*, *Rhizomucor*, or *L. corymbifera*. The experimental design is shown in Figure 2A, transferrin was serially diluted across the columns from 50 to 0 μg/mL, while LAMB was serially diluted across the rows from 0.4 to 0 μg/mL. This two-dimensional concentration matrix generated a series of unique transferrin–LAMB combinations. The final column, containing no transferrin, was used to assess LAMB alone, whereas the final row, containing no LAMB, was used to assess transferrin alone; the well containing neither agent served as the fungal growth control. This design allowed the inhibitory concentrations of each agent alone and in combination to be compared and used to calculate the fractional inhibitory concentration index (FICI). Representative images of fungal growth in the checkerboard assays are shown for *R. delemar*, *Rhizomucor*, and *L. corymbifera* in Figure 2B–D, respectively. Single-agent MICs of transferrin and LAMB were determined separately for each fungal isolate and are presented in Supplementary Figure S1. The MICs of each agent alone and the corresponding inhibitory concentrations in combination are summarized in Supplementary Table S1. Across all tested species, the combination of transferrin and LAMB inhibited fungal growth more effectively than either agent alone.

The calculated fractional inhibitory concentration index (FICI) values were <0.058 for *R. delemar*, <0.146 for *Rhizomucor*, and <0.058 for *L. corymbifera*, indicating strong synergistic interactions (FICI < 0.5). Notably, the concentration of LAMB required to inhibit fungal growth was substantially reduced when combined with transferrin. These results demonstrate that transferrin potentiates the antifungal activity of LAMB against multiple clinically relevant Mucorales species and supports its development as an adjunctive therapeutic strategy.

### 3.3. Transferrin Suppresses Expression of the CotH3 and GRP78

Because angioinvasion is a hallmark of mucormycosis pathogenesis, we next investigated whether transferrin influences the CotH3–GRP78 host–pathogen interaction that mediates endothelial invasion. *R. delemar* was co-incubated with HUVECs in the presence or absence of transferrin (10 μg/mL), and expression of fungal CotH3 and host GRP78 was quantified by qRT-PCR using the primers listed in Table 1.

**Figure 3:**
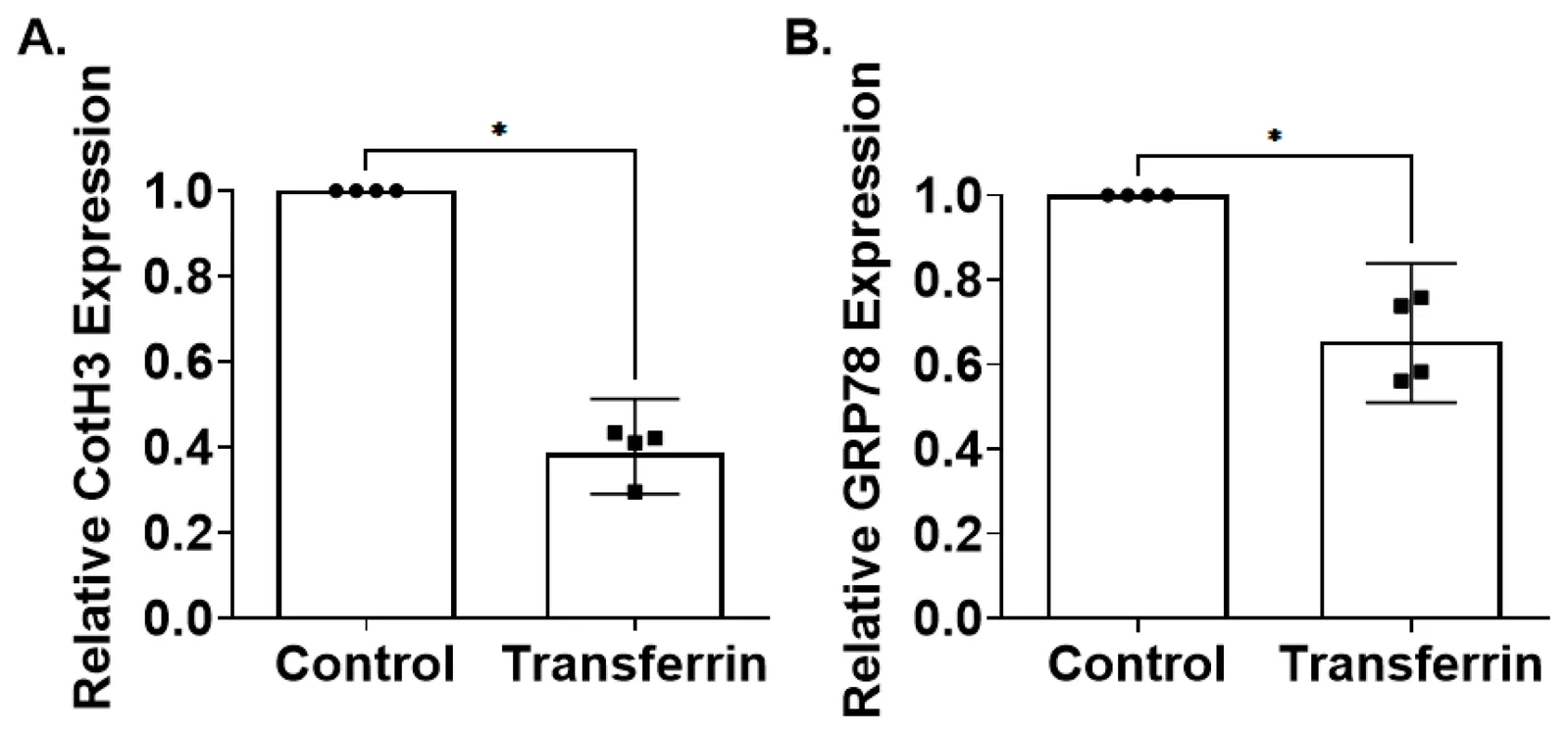
Transferrin reduces expression of fungal CotH3 and host GRP78 during fungal–endothelial co-culture. *R. delemar* was co-incubated with HUVECs in the presence or absence of transferrin (10 µg/mL) for 4 h. Total RNA was isolated separately from fungal and host cells, and expression of fungal CotH3 (**A**) and host GRP78 (**B**) was quantified by real-time PCR. Transferrin treatment significantly reduced expression of both CotH3 and GRP78. Data are presented as mean ± SD from four independent experiments. * Indicates *p* < 0.05.

Transferrin treatment significantly reduced expression of both fungal CotH3 and host GRP78 compared with untreated controls (Figure 3A,B). Specifically, transferrin reduced CotH3 expression in *R. delemar* by ~60%, suggesting that iron restriction suppresses expression of a key fungal ligand required for endothelial invasion. Concurrently, transferrin also decreased endothelial GRP78 expression by ~40%, indicating that transferrin may modulate both fungal and host determinants involved in the invasion process.

The CotH3–GRP78 interaction is a critical mediator of endothelial invasion during mucormycosis. Therefore, the observed reduction in expression of both components suggests that transferrin may modulate molecular determinants associated with endothelial invasion in addition to inhibiting fungal growth. Further studies will be required to determine whether these transcriptional changes translate into reduced endothelial invasion.

### 3.4. Transferrin Enhances the Therapeutic Efficacy of Antifungal Agents in Experimental Pulmonary Mucormycosis

Given the synergistic antifungal activity observed in vitro, we next evaluated whether transferrin could enhance the therapeutic efficacy of clinically relevant antifungal agents in vivo using an established neutropenic murine model of pulmonary mucormycosis. Neutropenic mice infected intratracheally with *R. delemar* were treated beginning 2 h after infection with transferrin alone or in combination with LAMB or ISAV. Antifungal doses were selected to provide suboptimal monotherapy activity, allowing the therapeutic contribution of adjunctive transferrin to be evaluated. LAMB was administered at 5 mg/kg intravenously once daily, whereas ISAV was administered orally at 78 mg/kg twice daily; the ISAV dose refers to the administered isavuconazonium sulfate prodrug rather than the active isavuconazole equivalent. Survival was monitored for 21 days.

**Figure 4:**
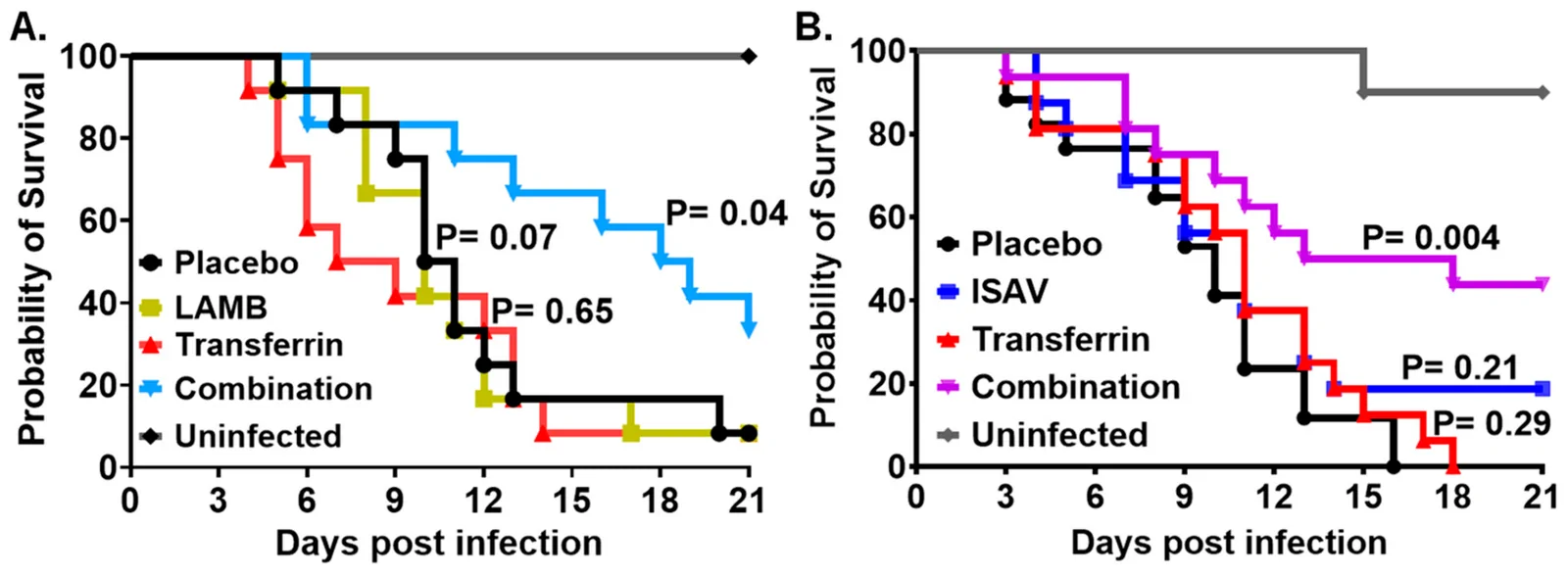
Transferrin enhances the therapeutic efficacy of clinically relevant antifungal agents in a neutropenic murine model of pulmonary mucormycosis. Neutropenic mice infected intratracheally with *R. delemar* were treated beginning 2 h post-infection and monitored for 21 days. (**A**) Survival of mice treated with placebo (*n* = 12), transferrin (90 mg/kg; *n* = 12), LAMB (5 mg/kg; *n* = 12), or transferrin plus LAMB (*n* = 12). The uninfected control group (*n* = 10) was included. Neither transferrin nor LAMB monotherapy significantly improved survival compared with placebo, whereas the transferrin plus LAMB combination significantly improved survival (*p* = 0.04). (**B**) Survival of mice treated with placebo (*n* = 17), transferrin (90 mg/kg; *n* = 16), ISAV (78 mg/kg; *n* = 16), or transferrin plus ISAV (*n* = 16). The uninfected control group (*n* = 10) was included. Neither transferrin nor ISAV monotherapy significantly improved survival compared with placebo, whereas the transferrin plus ISAV combination significantly improved survival (*p* = 0.004). Statistical significance was determined using the log-rank (Mantel–Cox) test.

In the LAMB study, transferrin alone did not improve survival compared with the placebo (untreated control) group (*p* = 0.65), while LAMB monotherapy showed a trend toward improved survival that did not reach statistical significance (*p* = 0.07) (Figure 4A). In contrast, the combination of transferrin and LAMB significantly improved survival compared with placebo (*p* = 0.04). Compared with placebo, the hazard ratios were 1.2 (95% CI, 0.52–2.8) for transferrin, 1.1 (95% CI, 0.48–2.6) for LAMB, and 0.42 (95% CI, 0.17–1.1) for transferrin plus LAMB. Thus, at the doses tested, a significant survival benefit was observed only with the combination treatment.

A similar pattern was observed in the ISAV study (Figure 4B). Neither transferrin alone (*p* = 0.29) nor ISAV monotherapy (*p* = 0.21) significantly improved survival compared with placebo. In contrast, the combination of transferrin and ISAV significantly improved survival compared with placebo (*p* = 0.004). Compared with placebo, the hazard ratios were 0.73 (95% CI, 0.36–1.44) for transferrin, 0.66 (95% CI, 0.32–1.40) for ISAV, and 0.35 (95% CI, 0.16–0.77) for transferrin plus ISAV. Together, these results demonstrate that adjunctive transferrin enhanced the therapeutic efficacy of two clinically relevant antifungal agents in experimental mucormycosis, with significant survival benefits observed for both combination regimens under conditions in which the corresponding monotherapies did not achieve statistical significance.

## 4. Discussion

Mucormycosis remains one of the most lethal invasive fungal infections despite the availability of effective antifungal agents [7]. In the present study, we demonstrate that transferrin possesses antifungal activity against multiple clinically relevant Mucorales fungi, reduces expression of fungal *coth3* and host *grp78*, and significantly enhances the therapeutic efficacy of both LAMB and ISAV in a neutropenic murine model of pulmonary mucormycosis due to *R. delemar* infection. Collectively, these findings provide preclinical evidence that transferrin modulates pathogenic determinants associated with mucormycosis and enhances the efficacy of current antifungal therapies, supporting further investigation of transferrin as a potential host-directed adjunctive treatment.

Iron acquisition is a well-established virulence determinant of Mucorales [15]. Previous studies have demonstrated that increased iron availability promotes fungal growth and pathogenicity, whereas iron restriction limits disease progression [10,28]. The importance of iron homeostasis is further highlighted by the increased susceptibility of patients with diabetic ketoacidosis and iron overload states to mucormycosis [15,29]. As the major physiological iron-binding protein in plasma, transferrin plays a central role in nutritional immunity by restricting extracellular iron availability and limiting microbial access to this essential nutrient [30,31]. Consistent with this role, our results demonstrate that transferrin inhibits the growth of multiple Mucorales species in a concentration-dependent manner, supporting the concept that iron sequestration contributes to host defense against these pathogens.

In addition to its effects on fungal growth, transferrin suppressed expression of both fungal CotH3 and host GRP78 during interactions between *R. delemar* and endothelial cells. The reduction in GRP78 expression should not be interpreted as an infection-specific effect of transferrin. In preliminary experiments, transferrin also reduced GRP78 expression in uninfected HUVECs (Figure S2), consistent with our previous finding that reduced iron availability decreases endothelial GRP78 expression [32]. Although a formal quantitative HUVEC viability assay was not performed, no obvious changes in cell morphology were observed following transferrin treatment under the conditions tested. The CotH3–GRP78 interaction is a critical mediator of endothelial invasion during mucormycosis and represents one of the best-characterized host–pathogen interactions in this disease [18,32]. Previous studies have demonstrated that disruption of this pathway reduces host cell invasion and improves outcomes in experimental mucormycosis [18,33]. Although the present study did not directly evaluate fungal invasion or vascular injury, the observed reduction in CotH3 and GRP78 expression indicates an association between transferrin treatment and altered expression of these invasion-associated factors. These findings do not establish that modulation of the CotH3-GRP78 pathway is responsible for the antifungal or survival benefit of transferrin. Further functional studies will be required to determine whether these transcriptional changes translate into reduced endothelial invasion or contribute to the therapeutic benefit observed in vivo.

One of the most notable findings of this study is the ability of transferrin to enhance the efficacy of clinically relevant antifungal therapies. Checkerboard assays demonstrated strong synergy between transferrin and LAMB against multiple Mucorales species. More importantly, this interaction translated into a significant survival benefit in vivo. Although neither transferrin nor LAMB monotherapy significantly improved survival at the doses tested, combination therapy significantly enhanced survival in infected neutropenic mice. These findings indicate that transferrin can potentiate antifungal efficacy under conditions in which individual treatments are insufficient to provide protection.

Importantly, the beneficial effects of transferrin were not limited to LAMB. Similar enhancement of therapeutic efficacy was observed when transferrin was combined with ISAV, an antifungal agent recommended in global guidelines for the treatment of mucormycosis [8,34]. The ability of transferrin to improve outcomes in combination with both a polyene and a triazole antifungal suggests that its activity is not dependent on a specific antifungal mechanism of action. Instead, transferrin may modify fundamental aspects of fungal physiology or host–pathogen interactions that increase susceptibility to antifungal therapy. This observation substantially broadens the potential translational relevance of transferrin as an adjunctive treatment approach. Because LAMB and ISAV were administered using different doses and treatment schedules, these experiments were not designed to directly compare the relative efficacy of the two combination regimens.

The precise mechanisms responsible for the enhanced therapeutic efficacy observed in vivo remain to be fully elucidated. Because transferrin inhibits fungal growth and reduces expression of fungal CotH3 and host GRP78, the observed survival benefit may reflect multiple complementary effects. Iron restriction may directly impair fungal metabolism and growth while also modulating virulence-associated pathways involved in host–pathogen interactions [15]. In addition, modulation of host responses to infection may contribute to improved outcomes. Future studies evaluating fungal burden, vascular injury, endothelial integrity, and host immune responses will be necessary to define the relative contribution of these mechanisms.

The therapeutic potential of iron restriction in mucormycosis must also be considered in the context of prior clinical experience with pharmacologic iron chelation [35]. In the randomized DEFEAT Mucor trial, adjunctive deferasirox combined with LAMB did not improve clinical outcomes compared with LAMB plus placebo. However, this phase II study enrolled only 20 patients and was complicated by substantial baseline imbalances between treatment groups, including differences in malignancy, neutropenia, corticosteroid use, and site of infection, limiting the extent to which these findings can be generalized to iron-restriction strategies more broadly. Importantly, transferrin is mechanistically distinct from small-molecule iron chelators such as deferasirox. Transferrin is an endogenous component of nutritional immunity that physiologically sequesters extracellular iron and restricts microbial access to this essential nutrient [36]. Consistent with this concept, Bruhn and Spellberg demonstrated that transferrin-mediated iron sequestration can enhance host defense against bacterial and fungal pathogens [37]. Thus, although the DEFEAT Mucor trial highlights the challenges of translating iron-targeted therapies to patients with mucormycosis, its findings do not necessarily predict the therapeutic potential of transferrin, which represents a biologically distinct strategy for limiting fungal access to iron.

The transferrin concentrations used in the in vitro experiments (up to 50 μg/mL) were substantially below normal circulating transferrin concentrations in humans (approximately 2000–3000 μg/mL) [38]. Thus, the concentrations exhibiting activity in vitro do not represent supraphysiological total transferrin concentrations. Nevertheless, circulating transferrin levels do not necessarily reflect the concentration or iron-saturation state of transferrin within infected tissues. Accordingly, the relationship between the concentrations exhibiting activity in vitro and therapeutically relevant transferrin exposure in vivo remains to be established.

This study has several limitations. First, the in vitro antifungal activity of transferrin was evaluated against only three Mucorales isolates/species. Although these organisms represent clinically relevant Mucorales, the present findings should not be generalized to all members of this order. Evaluation of a broader panel, including multiple clinical isolates within individual species, will be necessary to determine the spectrum and reproducibility of transferrin activity. Second, mechanistic analyses were limited to assessment of CotH3 and GRP78 expression, and direct measurements of endothelial invasion, vascular injury, or tissue fungal burden were not performed. As the present study was designed primarily as a proof-of-concept evaluation of therapeutic efficacy and survival, future studies will incorporate quantitative tissue fungal burden measurements to directly assess the effect of transferrin on fungal burden in vivo. Therefore, the observed reductions in CotH3 and GRP78 expression should not be interpreted as direct evidence of reduced endothelial invasion. Third, in vivo studies were conducted using a single experimental model of neutropenic pulmonary mucormycosis. Whether similar therapeutic benefits occur in other host backgrounds or anatomical forms of mucormycosis, including rhino-orbital, cutaneous, or gastrointestinal disease, remains unknown. In addition, transferrin treatment was initiated 2 h after infection, when the infecting organisms are expected to remain predominantly in the spore or early germination stage. In established mucormycosis, however, invasive hyphae represent the predominant tissue morphotype. Thus, the present study does not determine whether the protective effect of transferrin extends to established hyphal infection or whether part of its activity may result from effects on spore germination and early fungal growth. Future studies evaluating delayed transferrin administration after hyphal establishment will be important to better define its therapeutic potential in established disease. Although no overt treatment-related adverse effects were observed during routine monitoring, the present study was not designed as a formal safety or toxicology assessment, and systematic evaluation of body weight, clinical parameters, and treatment-related toxicity will be required in future studies. Finally, although transferrin-enhanced antifungal efficacy in combination with two clinically relevant agents, optimization of dosing strategies and pharmacologic parameters will be required before clinical translation can be considered.

In summary, we demonstrate that transferrin inhibits Mucorales growth, reduces expression of fungal CotH3 and host GRP78, and significantly enhances the efficacy of both LAMB and ISAV in experimental mucormycosis. These findings identify transferrin as a promising adjunctive therapeutic candidate and provide a foundation for future mechanistic and translational studies aimed at improving outcomes in this devastating fungal infection.

## 5. Conclusions

In conclusion, transferrin demonstrated antifungal activity against multiple clinically relevant Mucorales species, synergized with LAMB in vitro, and enhanced the therapeutic efficacy of both LAMB and ISAV in a neutropenic murine model of pulmonary mucormycosis. In addition, transferrin modulated expression of the CotH3–GRP78 host–pathogen pathway, suggesting that its activity may extend beyond inhibition of fungal growth alone. Importantly, the ability of transferrin to improve outcomes in combination with both a polyene and a triazole antifungal supports further preclinical investigation of transferrin as a potential adjunctive therapeutic approach for mucormycosis. Although the precise mechanisms underlying transferrin-enhanced protection remain to be fully elucidated, these findings provide a proof-of-concept that host iron sequestration can be leveraged to improve antifungal treatment efficacy and support further investigation of transferrin as a novel adjunctive therapy for invasive mucormycosis.

## Figures and Tables

**Table 1:** Primers used for quantitative real-time PCR (qRT-PCR) analysis.

Gene	Forward Primer (5′ → 3′)	Reverse Primer (5′ → 3′)
*C* *oth3*	GCCAATCCTAATGGTGAAGC	CATGAAACGGTCGAGATCAA
*actin*	AGCTCCTTTGAACCCCAAGT	ACGACCAGAGGCATACAAGG
*HSPA5* (GRP78)	GGAAAGAAGGTTACCCATGC	AGAAGAGACACATCGAAGGT
*GAPDH*	AGGCAACTAGGATGGTGTGG	TTGATTTTGGAGGGATCTCG

## Data Availability

All relevant data are within the manuscript and its Supplementary Materials. Requests for materials should be addressed to ibrahim@lundquist.org.

## Supplementary Materials

The following supporting information can be downloaded at https://www.mdpi.com/article/10.3390/jof12090682/s1, Figure S1: Single-agent antifungal activity of LAMB against Mucorales isolates; Figure S2: Effect of transferrin on GRP78 expression in uninfected HUVECs; Table S1: MICs and inhibitory concentrations of transferrin and LAMB alone and in combination against Mucorales isolates.
