# Supplementary material for: Transferrin Enhances Antifungal Therapy and Improves Survival in Experimental Mucormycosis
Source: J Fungi (Basel). 2026 Sep 11;12(9):682. doi: 10.3390/jof12090682 (PMC13608256; doi:10.3390/jof12090682)
Supplement: Supplementary file 1 [file jof-12-00682-s001.zip › jof-4555210-supplementary.pdf]

Supplementary Figures and Tables

Figure S1

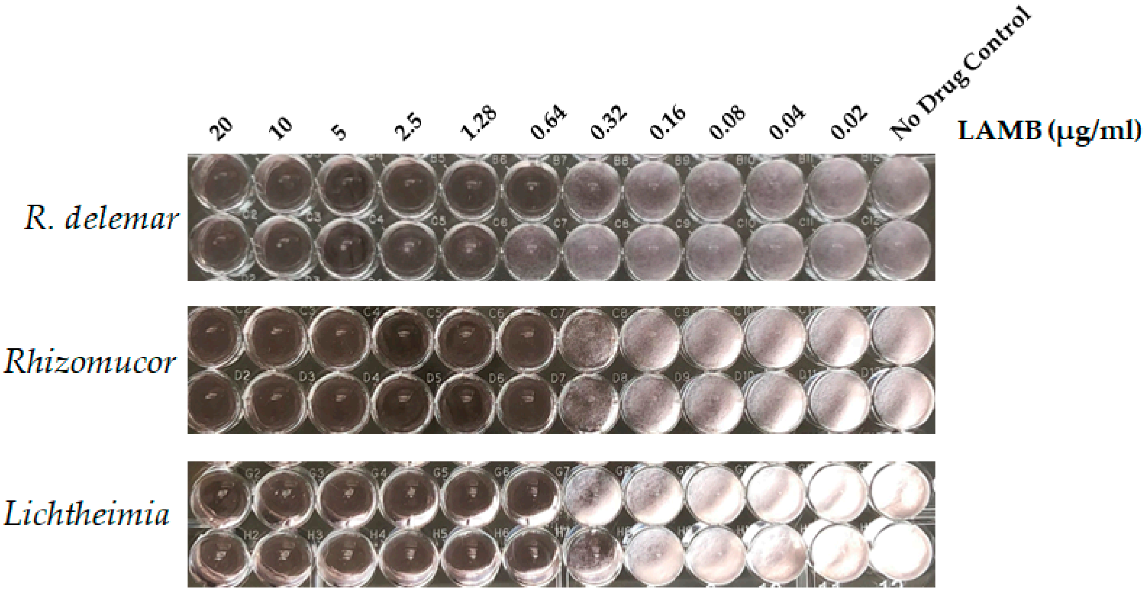

Figure S1: Single-agent antifungal activity of LAMB against Mucorales isolates. The minimum inhibitory concentrations (MICs) of liposomal amphotericin B (LAMB) were determined separately against *R. delemar*, *Rhizomucor* sp., and *L. corymbifera*. MICs were determined based on inhibition of fungal growth under the assay conditions described in the Materials and Methods.

Figure S2

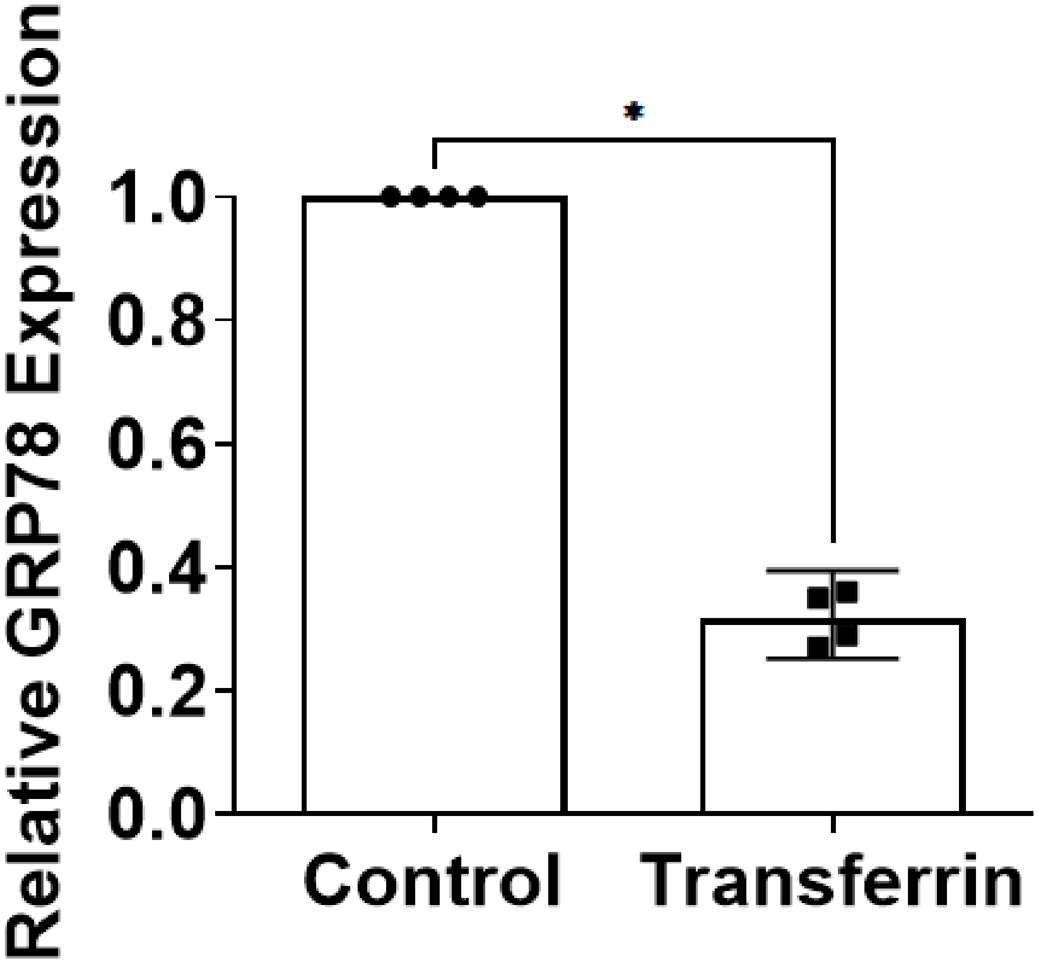

Figure S2: Effect of transferrin on GRP78 expression in uninfected HUVECs. Uninfected HUVECs were treated with transferrin, and GRP78 expression was assessed by RT-qPCR. Data are presented as GRP78 expression relative to untreated control HUVECs. Data are presented as mean ± SD from four independent experiments. * Indicates $p < 0.05$.

Table S1

Fungal isolate	Transferrin MIC alone (μg/mL)	LAMB MIC alone (μg/mL)	Transferrin in combination (μg/mL)	LAMB in combination (μg/mL)	FICI
R. delemar	>50	0.6	0.8	0.025	<0.058
Rhizomucor	>50	0.6	3.1	0.05	<0.1446
L. corymbifera	>50	0.6	0.8	0.025	<0.058

Table S1. MICs and inhibitory concentrations of transferrin and LAMB alone and in combination against Mucorales isolates. Single-agent minimum inhibitory concentrations (MICs) of transferrin and liposomal amphotericin B (LAMB), and the corresponding inhibitory concentrations of each agent in combination, are shown for each fungal isolate. The fractional inhibitory concentration index (FICI) was calculated as the sum of the FICs of transferrin and LAMB. Because the single-agent MIC of transferrin was >50 μg/mL, the calculated FICI values are reported as upper bounds. FICI values <0.5 were interpreted as synergy.
